# Exact solution to the problem of *N* bodies forming a multi-layer rotating structure

**DOI:** 10.1186/s40064-015-1141-1

**Published:** 2015-07-17

**Authors:** Joseph J Smulsky

**Affiliations:** Institute of Earth’s Cryosphere, Malygina Str. 86, PO Box 1230, 625000 Tyumen, Russia

**Keywords:** Problem of *N* bodies, Multi-layer rotating structure, Exact solution, Homographic and planar central configurations

## Abstract

Exact solutions to the problem of the Newtonian gravitational interaction of *N* material points moving around *N*_2_ concentric circular orbits are considered. Each circular orbit contains *N*_3_ axisymmetrically located bodies having identical masses. The structure as a whole rotates around its symmetry axis. Such structures are identical to the homographic-dynamics configurations, or planar central configurations, known from literature. Conceptually, those structures can be considered as structures formed by mutually embedded polygons with point bodies placed at polygon vortices. For structures involving less than 20 bodies, solutions were obtained using Hamiltonian-mechanics methods. In the study, the forces acting on each body in the rotating structure from the side of all other bodies were found. The differential motion equations of the bodies were reduced to a system of linear algebraic equations for the body masses. Solutions in various forms were obtained. For specifying the initial parameters and for calculating all other characteristics of the structures, a computer program RtCrcSt2.for has been developed. Structures comprising up to one million bodies have been calculated. Graphical images of obtained structures are presented, and their properties are described. Stability problems for examined structures are considered, and possible application of obtained results to celestial- and space-mechanics problems is discussed.

## Background

For some configurations of *N* material points, there exist exact solutions to the problem of the Newtonian gravitational interaction of the material points. For instance, for *N* bodies axisymmetrically arranged along a circumference, a complete solution to the problem was previously obtained in (Smulsky 1999, 2004; Smul’skii 2003). In this problem, a central body can be located at the center of the circumference. Depending on their initial velocities, the peripheral bodies can move along an ellipse, a parabola, or a hyperbola. Apart from such single-layer configurations, in a number of studies the matter of exact solutions for configurations involving several layers was addressed (Silushik 2003; Grebenikov et al. 2006; Diarova et al. 2006; Gutsu et al. 2007; Grebenikov 2010). Traditionally, the configurations of interest were treated as systems formed by mutually embedded polygons rotating, as an entity, at angular velocity *ω*. At polygon vertices, material points interacting among themselves by the Newton law of gravitation are placed. Within the context of the problem under consideration, example solutions for embedded triangles, rhombs, squares, pentagons, and hexagons were reported in a generalizing study by Grebenikov (Grebenikov 2010). The vertices in neighbor polygons can lie either in one radius or in radii passing through the middle points of the sides of neighbor polygons.

The above problem was solved for several layers of such polygons: for triangles, up to four layers; for squares, up to three layers, and for pentagons and hexagons, up to two layers (Grebenikov 2010). The largest number of the interacting bodies located at polygon vertices amounted to 12 bodies. Within the Hamiltonian dynamics, the problem can be reduced to a system of algebraic equations that can be solved by computer-algebra methods (Grebenikov 2010; Grebenikov et al. 2002). Each particular problem requires special consideration, and much effort has to be spent on its solution. Earlier, such problems for interacting material points were treated as homographic-dynamics problems (Grebenikov 2010) or problems of planar central configurations (Saari 1980; Perko and Walter 1985; Diacu 1990; Xia 1991; Albouy 1996; Bang and Elmabsout 2001; Moeckel 1990; Hampton and Moeckel 2006; Albouy et al. 2008; Shi and Xie 2010). In turn, homographic dynamics itself has emerged as a new field in space dynamics (Grebenikov 2010).

In the present publication, we consider a somewhat different approach to solving the problems described above. This approach presents further development of the method that was previously used in tackling the axisymmetric problem (Smulsky 1999, 2004; Smul’skii 2003) and in treating multi-layered ring structures (Smul’skii 2011). The motion of the bodies is investigated considering the forces acting between the bodies. Instead of polygons, whose imaginary sides connect the bodies, in the present study we deal with the circumferences along which the bodies are located. The authors of all the above-mentioned publications mainly studied theoretical aspects of the Hamiltonian dynamics of the systems of interest such as, for instance, the solution existence problem for a specific form of central configurations. Finding stable central configurations is very difficult a problem (Yu and Zhang 2015). The present work is devoted to obtaining all exact solutions, calculation of structures, and possible use of results obtained.

## Statement of the problem

**Figure 1:**
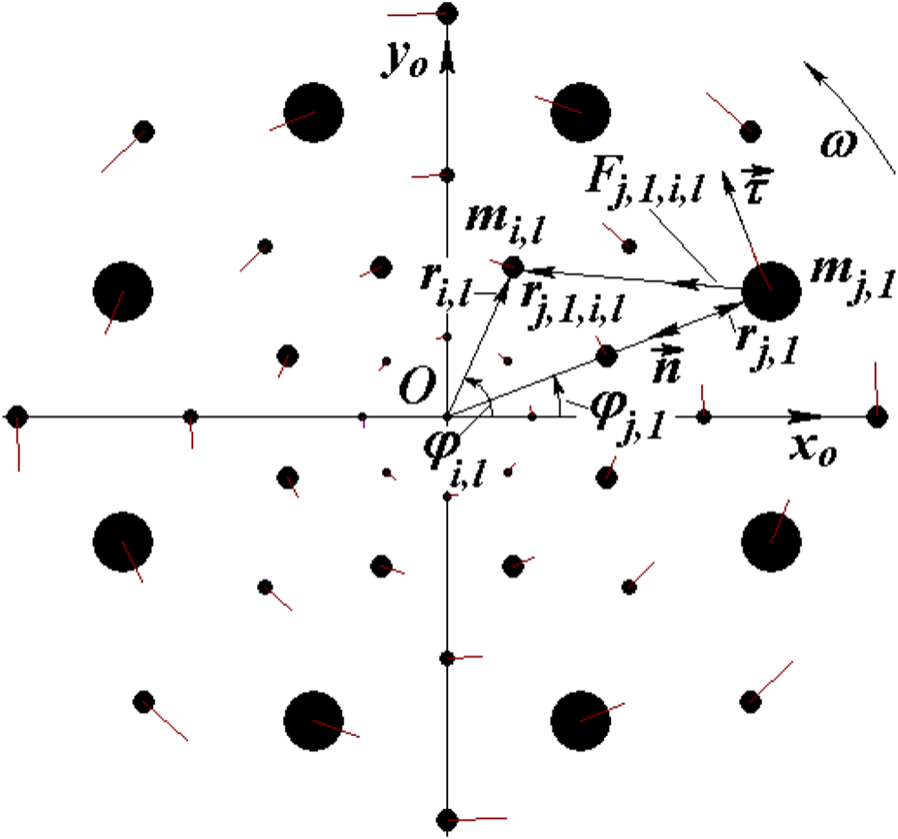
Geometric characteristics of the multi-layer axisymmetric structure with *N*_2_ = 5 and *N*_3_ = 8 in which the angles of the first body in neighbor rings *φ*_*j,*1_ alternate one another. For clarity, the bodies are shown as *full circles* whose radii vary in proportion to the bodies’ masses, and the line segments at the *circles* show the velocity vectors of the bodies.

Consider a multi-layer axisymmetric structure comprising several material points whose interaction is governed by the Newton law of gravitation (see Figure 1). The structure involves *N*_2_ circumferences with *N*_3_ bodies located in each circumference. We will call the system of bodies whose centers are arranged along a circumference a body ring, or a body layer. The rings are enumerated with numbers *j* = 1, 2 … *N*_2_, and the bodies in each ring, with numbers *l* = 1, 2 … *N*_3_. In the plane *x*_*o*_*y*_*o*_, in which the bodies are disposed, for a body of mass *m*_*j,l*_ we introduce a polar radius *r*_*j,l*_ and a polar angle *φ*_*j,l*_. For simplicity, in what follows the symbol *m*_*j,l*_ will also be used to denote the body itself. All bodies in a ring have identical radii *r*_*j,l*_ = *r*_*j*_, where *r*_*j*_ is the radius of the ring, and their masses are also identical, i.e. *m*_*j,l*_ = *m*_*j*_. The polar angle that defines the angular position of the first body in each ring, *φ*_*j,*1_, specifies the particular form of the structure. In what follows, this angle will be assumed a specified parameter. The polar angles of all other peripheral bodies are given by the formula

1 $$\varphi_{j,l} = \varphi_{j,1} + (l - 1) \cdot \Delta \varphi_{0} ,$$

where *Δφ*_0_ = 2*π*/*N*_3_ is the angular separation between the bodies in each ring.

To summarize, the geometry of a multi-layered axisymmetric structure is defined by the total number of the involved rings *N*_2_, by the number of the bodies in each ring *N*_3_, by the ring radii *r*_*j*_, and by the angles defining the angular position of the first body in each ring *φ*_*j,*1_. The mass of each body in the *j*-th ring is *m*_*j*_ and, in the presence of a central body of mass *m*_0_, the mass of the whole system is

2 $$m_{SS} = m_{0} + N_{3} \cdot \sum\limits_{j = 1}^{{N_{2} }} {m_{j} }$$

The whole system revolves at angular velocity *ω*. A multi-layer structure is specified by the set quantities *N*_2_, *N*_3_, *φ*_*j,*1_, *m*_0_ and *ω*, while the ring radii *r*_*j*_ and the body masses *m*_*j*_ present unknown quantities.

## The forces acting between the bodies

Consider the forces that act on the first body in the *j*-th ring, whose mass is *m*_*j,*1_, from the side of all other bodies (see Figure 1). To the body *m*_*j,*1_, we attach a natural coordinate system (*n*,*τ*) in which *n* and *τ* are the normal and tangent lines to the trajectory. The gravity force *F*_*j*,1,*i*,*l*_ due to the body *m*_*i,l*_ in the *i*-th ring that acts on the body *m*_*j,*1_ is *F*_*j*,1,*i*,*l*_ = *G*·*m*_*j*,1_·*m*_*i*,*l*_/*r*_*j*,1,*i*,*l*_^*2*^, where *G* is the gravitation constant and *r*_*j*,1,*i*,*l*_ is the distance from body *m*_*j*,1_ to body *m*_*i*,*l*_. Then, the projections of the force *F*_*j*,1,*i*,*l*_ onto the axes $\vec{n}$ and $\vec{\tau }$ are

3 $$F_{n,j,1,i,l} = \frac{{G \cdot m_{j,1} \cdot m_{i,l} \cdot n_{j,1,i,l} }}{{r_{j,1,i,l}^{3} }};$$

4 $$F_{\tau ,j,1,i,l} = \frac{{G \cdot m_{j,1} \cdot m_{i,l} \cdot \tau_{j,1,i,l} }}{{r_{j,1,i,l}^{3} }},$$

with *n*_*j*,1,*i*,*l*_ and *τ*_*j*,1,*i*,*l*_ being the projections of the distance *r*_*j*,1,*i*,*l*_, respectively onto the *n*- and *τ*-axis.

In the triangle *Om*_*i,l*_*m*_*j*,1_ (see Figure 1), the angle between the body radii *r*_*i*_ and *r*_*j*_ is

5 $$\Delta \varphi_{j,1,i,l} = \varphi_{i,l} - \varphi_{j,1} .$$

By the cosine theorem, the distance between the bodies is

6 $$r_{j,1,i,l}^{2} = r_{j}^{2} + r_{i}^{2} - 2r_{i} r_{j} \cdot \cos \Delta \varphi_{j,1,i,l} .$$

Then, the projections of the latter distance onto the *n*- and *τ*-axis are respectively

7 $$n_{j,1,i,l} = - (r_{i}^{{}} \cdot \cos \Delta \varphi_{j,1,i,l} - r_{j} );\quad \tau_{j,1,i,l} = r_{i}^{{}} \cdot \sin \Delta \varphi_{j,1,i,l} .$$

Apart from the peripheral bodies, the body *m*_*j*_ is additionally acted upon by the central body of mass *m*_0_ located at the point *O* (see Figure 1). The projection of the force due to the latter body onto the *τ*-axis is zero, and the projection of that force onto the *n*–axis can be expressed by analogy with formula (3):

$$F_{n,j,1,0} = \frac{{G \cdot m_{j,1} \cdot m_{0} \cdot n_{j} }}{{r_{j}^{3} }}.$$

In view of formula (7), in which we assume *r*_*i*_ = 0, for the central body the latter formula yields *n*_*j*_ = *r*_*j*_.

After substitution of (6) and (7) into expressions (3) and (4), and after summation of the forces over all bodies in the system, for the projections of the forces acting on the body *m*_*j*,1_ from the side of the rest bodies we obtain:

8 $$F_{n,j,1} = G \cdot m_{j} \left[ {\frac{{m_{0} }}{{r_{j}^{2} }} + \sum\limits_{i \ne j}^{{N_{2} }} {\left[m_{i} \cdot \sum\limits_{l = 1}^{{N_{3} }} {\frac{{r_{j} - r_{i} \cdot \cos \Delta \varphi_{j,1,i,l} }}{{\left( {r_{j}^{2} + r_{i}^{2} - 2r_{j} \cdot r_{i} \cdot \cos \Delta \varphi_{j,1,i,l} } \right)^{3/2} }}} \right]} + \frac{{m_{j} }}{{r_{j}^{2} }}\sum\limits_{l = 2}^{{N_{3} }} {\frac{0.5}{{\left( {2 - 2 \cdot \cos \Delta \varphi_{j,1,j,l} } \right)^{1/2} }}} } \right];$$

9 $$F_{\tau ,j,1} = G \cdot m_{j} \left[ {\sum\limits_{i \ne j}^{{N_{2} }} {\left[m_{i} \cdot \sum\limits_{l = 1}^{{N_{3} }} {\frac{{r_{i} \cdot \sin \Delta \varphi_{j,1,i,l} }}{{\left( {r_{j}^{2} + r_{i}^{2} - 2r_{j} \cdot r_{i} \cdot \cos \Delta \varphi_{j,1,i,l} } \right)^{3/2} }}} \right]} + \frac{{m_{j} }}{{r_{j}^{2} }}\sum\limits_{l = 2}^{{N_{3} }} {\frac{{\sin \Delta \varphi_{j,1,i,l} }}{{\left( {2 - 2 \cdot \cos \Delta \varphi_{j,1,j,l} } \right)^{3/2} }}} } \right]$$

In (8) and (9), *m*_*j*_ is mass of each of the bodies *m*_*j*,1_, and *m*_*i*_ is the mass of each of the bodies *m*_*i*,*l*_.

For excluding the self-action force of the body *m*_*j*,1_, in formulas (8) and (9) the action due to all other bodies in the *j*-th ring is separated out from the total sum, and that action is then written as a last term. This action readily results if we replace the subscript *i* in the previous term with the subscript *j*. The exclusion of the *j*-th ring from the expression under the summation sign $\sum {}$ is denoted as *i* ≠ *j*.

We consider such configurations of rotating structures for which expressions (8) and (9) yield one and the same force value for each body in the *j*-th ring. The latter is only possible if, as the *n*-axis passes through any body in the *j*-th ring, the geometric positions of all other bodies acting on this body remain unchanged. The latter condition is satisfied providing that the initial angle of ring bodies assumes either a value *φ*_*j*,1_ = 0 or a value *φ*_*j*,1_ = 0.5·*Δφ*_0_. The arrangement of a structure in which the initial angles *φ*_*j*,1_ assume sequentially alternating values in neighbor rings is shown in Figure 1. Structures with arbitrary alternation pattern of initial angles *φ*_*j*,1_ also satisfy the above condition.

Note that it is the above conditions that define the term «axisymmetric» as used in the present article. Some structure presents an axisymmetric structure if its geometric and dynamic characteristics remain unchanged as the structure revolves through the angle *Δφ*_0_.

For the configurations considered above, the normal *n* presents a symmetry axis (see Figure 1). That is why, in view of (5), the angles of deflection of the interacting bodies from the *n*-axis, *Δφ*_*j*,1,*i*,*l*_, have values pairwise equal in magnitude and opposite in sign. Hence, the sines in the nominators of formula (9) are also pairwise equal in magnitude and opposite in sign. The cosines of those angles in the denominators being identical, the tangential forces vanish. With the total number of the bodies *N*_3_ being an even number, another body can be found in the *n*-axis, this body being located symmetrically about the center *O*. Since the angle *Δφ*_*j*,1,*i*,*l*_ of the latter body is *π*, then the interaction force due to this body in (9) is also zero. Thus, the projections of all the forces onto the tangent axis are zero, $F_{\tau ,j,1} = 0$. That is why the force acting on each body in the ring with number *j* from the side of all other bodies in the multi-layer axisymmetric structure is directed along the normal *n* to the trajectory, i.e. towards the center *O*, and this force is defined by expression (8).

According to (1), the difference between the angles (5) in the *j*-th ring is

10 $$\Delta \varphi_{j,1,j,l} = \varphi_{j,l} - \varphi_{j,1} = 2\pi (l - 1)/N_{3} .$$

Then, the expression in the denominator of the last term in formula (8) can be written as

11 $$2\cdot [ 1- { \cos }( 2\pi (l - 1)/N_{ 3} )] = 4\cdot { \sin }^{ 2} (\pi (l - 1)/N_{ 3} ).$$

After substitution of (11) into (8), for the force acting on arbitrary body in the *j*-th ring from the side of all other bodies we obtain the expression

12 $$F_{{n,j}} = \frac{{G \cdot m_{j} }}{{r_{j}^{2} }}\left[ {m_{0} + \sum\limits_{{i \ne j}}^{{N_{2} }} {\left[ {m_{i} \cdot \sum\limits_{{l = 1}}^{{N_{3} }} {\frac{{1 - r_{{i,j}} \cdot \cos \Delta \phi _{{j,1,i,l}} }}{{\left( {1 + r_{{i,j}}^{2} - 2r_{{i,j}} \cdot \cos \Delta \phi _{{j,1,i,l}} } \right)^{{3/2}} }}} } \right]} + m_{j} \cdot f_{{n3}} } \right]$$

where

13 $$f_{n3} = 0.25\sum\limits_{l = 2}^{{N_{3} }} {\frac{1}{{\sin \left[ {\pi (l - 1)/N_{3} } \right]}}} ;$$

*r*_*i,,j*_ = *r*_*i*_/*r*_*j*_ is the ratio between the radii of the rings with numbers *i* and *j*; and the angle difference *Δφ*_*j*,1,*j*,*l*_ is given by formula (10).

Force (12) is directed towards the center *O*.

## Motion equations of the rotating structure

By the force (12), the body *m*_*j*,1_, whose mass is *m*_*j*_, executes an accelerated motion (see Figure 1). In the natural coordinate system (*n*, *τ*), the force (12) acts along the *n*-axis; along the same axis, the normal acceleration *w*_*n*_ = *v*^*2*^*/ρ* is directed (here, *v* is the tangential velocity of the body *m*_*j*,1_, and *ρ* is the radius of curvature of the body’s trajectory. Hence, the differential equation of motion for the body can be written as

14 $$m_{j} \cdot \frac{{v^{2} }}{\rho } = F_{n,j} .$$

We consider a rotating structure revolving with angular velocity *ω* at fixed orbital radii. Hence, for the body *m*_*j*,1_ the radius of curvature of its trajectory is *ρ* = *r*_*j*_, and the velocity of the body is *v* = *ω*·*r*_*j*_. After substitution of the above quantities and force (12) into Eq. (14), the differential motion equation of the body *m*_*j*,1_ acquires the form

15 $$\omega^{2} = \frac{G}{{r_{j}^{3} }}\left[ {m_{0} + \sum\limits_{i \ne j}^{{N_{2} }} {\left[m_{i} \cdot \sum\limits_{l = 1}^{{N_{3} }} {\frac{{1 - r_{i,j} \cdot \cos \Delta \varphi_{j,1,i,l} }}{{\left( {1 + r_{i,j}^{2} - 2r_{i,j} \cdot \cos \Delta \varphi_{j,1,i,l} } \right)^{3/2} }}}\right] + m_{j} \cdot f_{n3} } } \right],$$

where *j* = 1, 2, … *N*_2_.

Thus, the motion of the bodies making up a rotating structure is governed by *N*_2_ Eq. (15). This system of equations is an algebraic system. As it was noted above, the unknown quantities here are the ring radii *r*_*j*_ and the body masses *m*_*j*_. With the ring radii *r*_*j*_ specified, the body masses *m*_*j*_ are defined by Eq. (15). If necessary, it is the body masses *m*_*j*_ that can be specified; then, the ring radii *r*_*j*_ can be found from Eq. (15).

For generating a uniform algorithm for solving the problem for multi-layer structures with and without a central body, we introduce an initial total mass *m*_*in*_ of the central body and all the peripheral bodies forming the first ring. We denote the mass fraction due to the central body in this structure as *p*_*m0*_. Then, the mass of the central body is *m*_0_ = *m*_*in*_*∙p*_*m0*_. A structure without a central body is defined by the equality *p*_*m0*_ = 0.

An analysis of potential applications of this problem shows that a good strategy towards solving the problem consists in specification of the geometry of the multi-layer axisymmetric structure, including the ring radii *r*_*j*_, followed by the determination of the masses *m*_*j*_ (via solving system (15)). We therefore rewrite Eq. (15) in a different form. We isolate in the second term the non-dimensional acceleration of the body *m*_*j*,1_ due to the action of a unit mass contained in the body *m*_*i*,1_ of the *i*-th ring:

16 $$Q_{j,1,i,l} = \frac{{1 - r_{i,j} \cdot \cos \Delta \varphi_{j,1,i,l} }}{{\left( {1 + r_{i,j}^{2} - 2r_{i,j} \cdot \cos \Delta \varphi_{j,1,i,l} } \right)^{3/2} }},$$

where *r*_*i,,j*_ = *r*_*i*_/*r*_*j*_ is the non-dimensional ratio of the radii.

Then, the non-dimensional acceleration of the body *m*_*j,1*_ due to the interaction of that body with all the bodies in the *i*-th ring is

17 $$a_{j,i} = \sum\limits_{l = 1}^{{N_{3} }} {Q_{j,1,i,l} } .$$

Since the bodies forming the ring impart each body in the same ring with a non-dimensional acceleration *f*_*n*3_, we introduce a designation

18 $$a_{j,j} = f_{n 3} .$$

We normalize the masses of all the bodies by the initial mass *m*_*in*_, and denote the non-dimensional masses of the bodies as

19 $$m_{ud,j} = m_{j} /m_{in}; \quad m_{ud,0} = m_{0} /m_{in} .$$

Then, using formulas (16)–(19), we can rewrite Eq. (15) as the following system of linear algebraic equations:

20 $$\sum\limits_{i = 1}^{{N_{2} }} {a_{j,i} \cdot m_{ud,i} } = b_{j} ,\quad j = 1,2 \ldots N_{2} ,$$

where

21 $$b_{j} = c_{i} \cdot r_{ud,j}^{ 3} - m_{ud,0} ;\quad r_{ud,j} = r_{j} /r_{1} ;\quad c_{i} = r_{ 1}^{ 3} \omega^{ 2} /(m_{in} \cdot G).$$

In the system of linear algebraic Eq. (20), the specified parameters are *N*_2_, *N*_3_, *φ*_*j,*1_, *m*_*in*_, *p*_*m0*_, *ω*, and *r*_*j*_, and the unknown quantities are the non-dimensional masses *m*_*ud,i*_ of the peripheral bodies. The mass of the central body remains unchanged, i.e. *m*_0_ = const. On the other hand, the non-dimensional masses of all the peripheral bodies *m*_*ud,j*_ result from the solution of Eq. (20).

## Solution of the equations

The solution of linear algebraic equation system (20) is given (Korn and Korn 1968) by

22 $$m_{ud,i} = D_{i} /D,$$

where *D* is the determinant of the matrix *a*_*j*,*I,*_

23 $$D = { \det }\left[ {a_{j,i} } \right],$$

and *D*_*i*_ is the determinant of the matrix *a*_*j*,*i*_ in which the *i*-th column is replaced with the free-term column *b*_*i*_.

In the case of two rings, we have $$D = \left| {\begin{array}{*{20}c} {a_{11} } & {a_{12} } \\ {a_{21} } & {a_{22} } \\ \end{array} } \right| = a_{11} a_{22} - a_{21} a_{12}$$ and, also, $$D_{1} = \left| {\begin{array}{*{20}c} {b_{1} } & {a_{12} } \\ {b_{2} } & {a_{22} } \\ \end{array} } \right| = b_{1} a_{22} - b_{2} a_{12}$$ and $$D_{2} = \left| {\begin{array}{*{20}c} {a_{11} } & {b_{1} } \\ {a_{22} } & {b_{2} } \\ \end{array} } \right| = a_{11} b_{2} - a_{22} b_{1} .$$ Then, in view of (22), we can write the solutions of Eq. (20) for the masses of one body in the first and second layer as

24 $$m_{ud,1} = (b_{1} a_{22} - b_{2} a_{12} )/(a_{11} a_{22} - a_{21} a_{12} );\quad m_{ud,2} = (a_{11} b_{2} - a_{21} b_{1} )/(a_{11} a_{22} - a_{21} a_{12} )$$

Solutions (24) define a rotating structure formed by two layers. In each layer, there are *N*_3_ bodies, where *N*_3_ is an arbitrary integer number. The total number of the bodies in such a two-layer structure is *N* = 2*N*_3_ + 1.

In the case of three or a greater number of layers, the solutions of system (20) emerge as too cumbersome expressions hard to be used for calculating the non-dimensional masses *m*_*ud,i*_. Specification of a large number of initial parameters also becomes difficult. Moreover, the solution of the linear system of Eq. (20) may yield negative masses *m*_*ud,i*_. That is why variation of initial parameters of the rotating system has to be applied to obtain positive values for all masses. For a greater number of rings *N*_2_ and for a greater number of bodies in the rings *N*_3_ the latter leads to a greater amount of computations. For executing the computations, several computers programs have been developed. It was found that the above problems can be solved most easily via numerical solution of the algebraic equation system (15) or (20). The computer programs were written in the FORTRAN language (Smulsky 2013a). In the first solution version of the problem in the program RtCrcStr.for, the equation system (15) was solved by the iteration method. In the second solution version of the problem in the program RtCrcSt2.for, for solving the equation system (20) the Gauss method was used. The latter version being simpler, below we consider this version. Nevertheless, it should be noted here that, at certain *N*_2_-to-*N*_3_ ratios, it is easier to get a solution of equations using RtCrcStr.for.

The initial data for a particular rotating structure are specified in the file RtCrcSt2.dat. Instead of the angular velocity *ω*, we deal with the structure’s rotational period *P*_*rd*_ = 2*π*/*ω*. In the program, the distances and the times are used as relative quantities. That is why the period *P*_*rd*_ is specified in sidereal years. The ring radii are specified in the data file RtCrcSt2.dat with the help of a new parameter *o*_*kr*_ defined by the relation

25 $$r_{j} = j \cdot o_{kr} \cdot r_{ 1} ,$$

where *r*_1_ is the radius of the first ring. The value of *o*_*kr*_ cannot be smaller than 0.5.

The radius of the first ring can be determined from the existence condition of this ring at some specified mass of the central body *m*_0_ and at some initial body mass *m*_1_ in the first ring (Smulsky 1999, 2004; Smul’skii 2003). The latter condition results from motion Eq. (15) on disregard of other rings:

26 $$r_{1} = \left[ {\frac{{G(m_{0} + m_{1} \cdot f_{n3} )}}{{\omega^{2} }}} \right]^{1/3} .$$

The angles that define the position of the first bodies *φ*_*j*,1_ can be specified in two ways: (1) all angles *φ*_*j*,1_ = 0 and (2) the angle *φ*_*j*,1_ = 0 alternates with the angle *φ*_*j*,1_ = 0.5∙*Δφ*_0_. Other patterns of the radii *r*_*j*_ and angles *φ*_*j*,1_ can be specified in additional files that can be included in the data file RtCrcSt2.dat.

The executable module of the program RtCrcSt2.exe generates several output data files, including a special file with specified values of all kinematic parameters of the structure. That file contains initial data and conditions for the program Galactica (Smulsky 2012a). In Galactica, a high-precision method for numerical integration of differential motion equations for material points interacting with one another by the Newton law of gravitation is implemented. The system Galactica is available in free access (Smulsky 2012b, c). This system computes the dynamics of the structures made up by interacting material points, and it allows one to investigate the evolution of such structures in time.

All structures obtained with the help of the program RtCrcSt2.exe were verified using the program Galactica. It should be noted here that Galactica integrates non-simplified equations, not Eqs. (15) or (20). The program Galactica contains an option allowing graphical representation of treated systems. Below, we reproduce the displayed images of several structures obtained after the first step in integration of their differential motion equations.

A description of the program RtCrcSt2.for and its functioning algorithm, and also the listing of the program, can be found elsewhere (Smulsky 2013a). The executable files are available at http://www.ikz.ru/~smulski/Data/RtCrcStr/.

## Examples of rotating structures

In the present study, multi-layer rotating structures involving 1, 2, 3, 4, 5, 15, 30, 100, 103 and 1,000 rings have been calculated. In the rings, the numbers of the bodies, 2, 5, 8, 10, 29, 30 and 999, were specified parameters. The total number of the bodies reached one million. Configurations with various values of the angle of the first body *φ*_*j,*1_ in the rings, and also with various alternation patterns of the angles in neighbor rings, were treated. Structures with different starting masses were calculated. As the initial masses, the Sun’s and Earth’s masses were set. Structures with and without a central body were considered.

A structure involving five rings with each of the rings comprising eight bodies (*N*_2_ = 5 and *N*_3_ = 8) with rotational period *P*_*rd*_ = 1 year is shown in Figure 1. The numbers of the rings are counted from the center *O*, and the numbers of the bodies, from the *x*_*O*_-axis. The angles *φ*_*j,*1_ of the first bodies in the rings sequentially assume values 0, *Δφ*_0_/2, 0, *Δφ*_0_/2 and 0. The initial mass *m*_*in*_ = 1.98912 × 10^30^ kg was specified to be somewhat heavier than the Sun’s mass. For the ring radii and for the masses of one body in the rings normalized, respectively by the radius of the first ring and by the mass of one body in the first ring, the values *r*_*ud,j*_ = 0, 1, 2.005, 2.985, 4.082, and 4.980, and *m*_*ud1,j*_ = *m*_*j*_/*m*_*1*_ = 1.256, 1, 2.957, 2.250, 7.712 and 2.973 were obtained. Here, the ring with radius $r_{ud,0}$ = 0 represents the central body. In the calculated structure, the radius of the first ring and the mass of one body in this ring are respectively *r*_*1*_ = 1.481 × 10^11^ m and *m*_*1*_ = 1.568 × 10^30^ kg. The mass of the whole structure is *m*_*SS*_ = 2.138 × 10^32^ kg, this mass being 108 times greater than the Sun’s mass.

**Figure 2:**
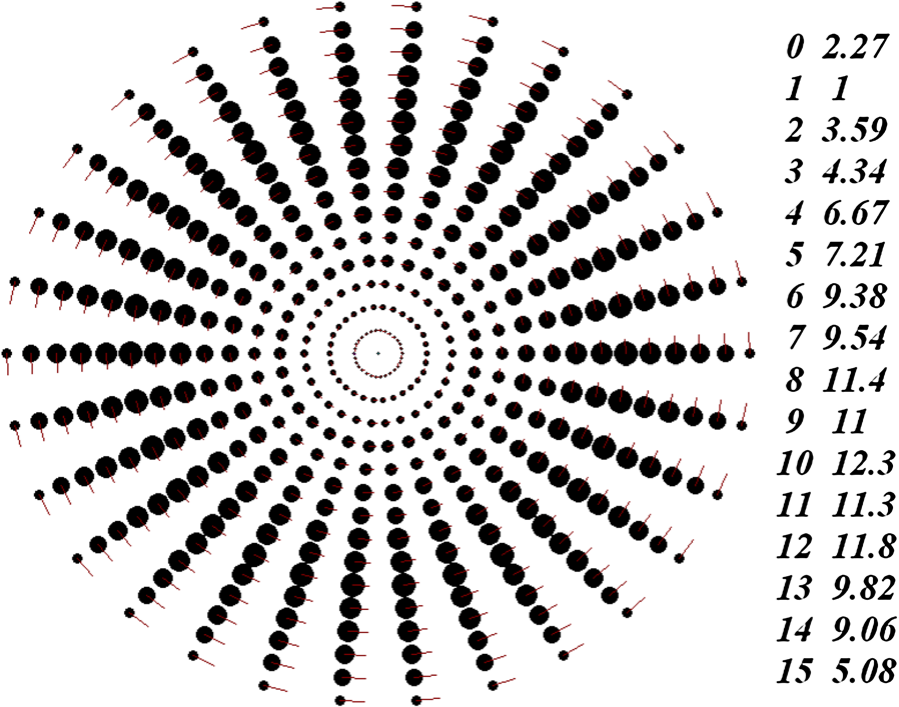
Image of a multi-layer axisymmetric rotating structure as seen on PC display. The characteristics of the structure were obtained by integration of differential motion equations performed by the program Galactica during one time step: *N*_2_ = 15, *N*_3_ = 30, *φ*_*j,*1_ = 0, *P*_*rd*_ = 1 year; the mass of the central body is equal to the Sun’s mass. Additionally, the number columns in the figure show the ring radii and the masses of one body in the rings normalized, respectively by the radius of the first ring *r*_*1*_ = 1.493837 × 10^11^ m and by the mass of one body in the first ring *m*_*1*_ = 8.684966 × 10^29^ kg. The total mass of the whole structure is *m*_*SS*_ = 3.218489 × 10^33^ kg.

Consider now three structures comprising fifteen rings (*N*_2_ = 15) with thirty bodies contained in each of the rings (*N*_3_ = 30); those structures have different alternation patterns of the angles *φ*_*j,*1_ of the first body in the rings. The rotating structure with zero angles of the first body in all rings, *φ*_*j,*1_ = 0, is shown in Figure 2. The line segments at the peripheral bodies indicate their velocity vectors.

As it is seen from Figure 2, the structure possesses a radial-beam configuration. In homographic dynamics (Grebenikov 2010), that structure would be termed the configuration comprising 15 concentrically embedded equilateral 30-gons. From the second number column in Figure 2, we see that the least masses belong to bodies in the first ring. Providing that the mass of the central body *m*_*0*_ is equal to the Sun’s mass, the mass *m*_*1*_ appears to be 2.27 times lighter than the mass *m*_*0*_. On going from the first to tenth ring, the masses of the bodies show almost a monotonic increase and, afterwards, they decrease in value. In that structure, the heaviest mass of the central body is *m*_*10*_ = 12.3·*m*_*1*_.

In the structure shown in Figure 2 and in two subsequent structures, the ring radii increase in value due to the addition of the radius of the first ring *r*_*1*_ (see Eq. (25)). In the structure shown in Figure 1, the ring radii show a different variation pattern. The latter can be attributed to the fact that, in the latter case, a proportional increase of ring radii results in an emergence of negative masses in the solution of Eq. (20). It should be noted that the structure shown in Figure 1 was obtained using the program RtCrcStr.for.

**Figure 3:**
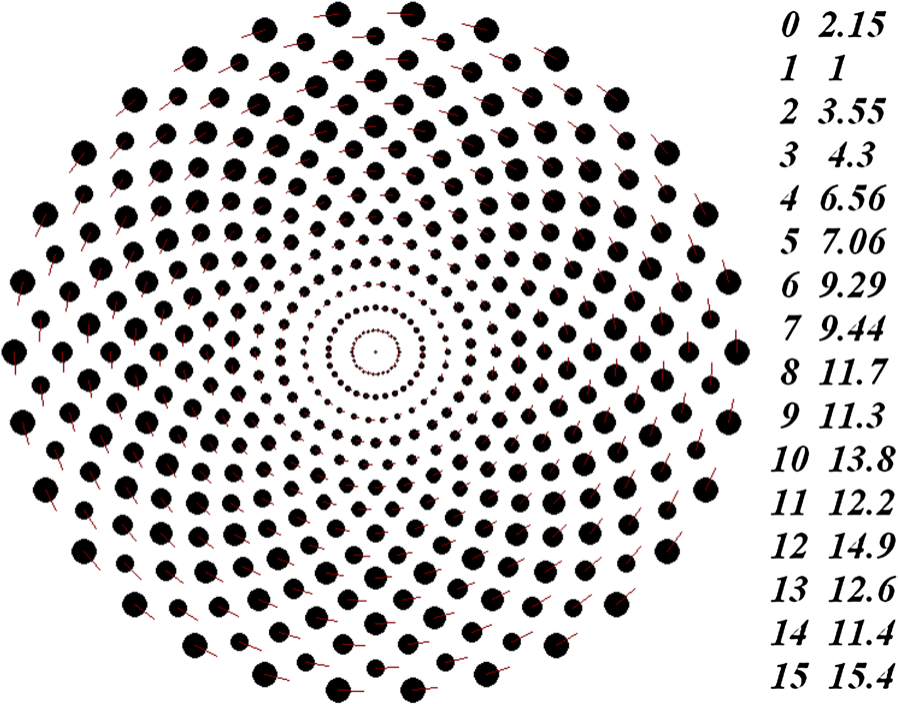
Multi-layer axisymmetric structure with *N*_2_ = 15 and *N*_3_ = 30; the angle *φ*_*j,*1_ = 0 alternates with the angle *φ*_*j,*1_ = 0.5·*Δφ*_0_; *P*_*rd*_ = 1 year; *r*_*1*_ = 1.493837 × 10^11^ m; *m*_*1*_ = 9.172058 × 10^29^ kg and *m*_*SS*_ = 3.977302 × 10^33^ kg. The rest designations are the same as in Figure 2.

Figure 3 shows a multi-layer rotating structure with sequential alternation of the angles *φ*_*j,*1_ of the first body in the rings. In homographic dynamics (Grebenikov 2010), this structure would be termed a configuration formed by 15 mutually embedded equilateral 30-gons rotated with respect to each other through the angle π/15. In comparison with the structure shown in Figure 2, the total mass of this structure has increased by a factor of 1.236. Here, the masses of the bodies in the rings also increase in value starting from the center. Nonetheless, the monotonic increase of the masses is violated after the tenth ring, and the heaviest bodies occur in the last ring.

**Figure 4:**
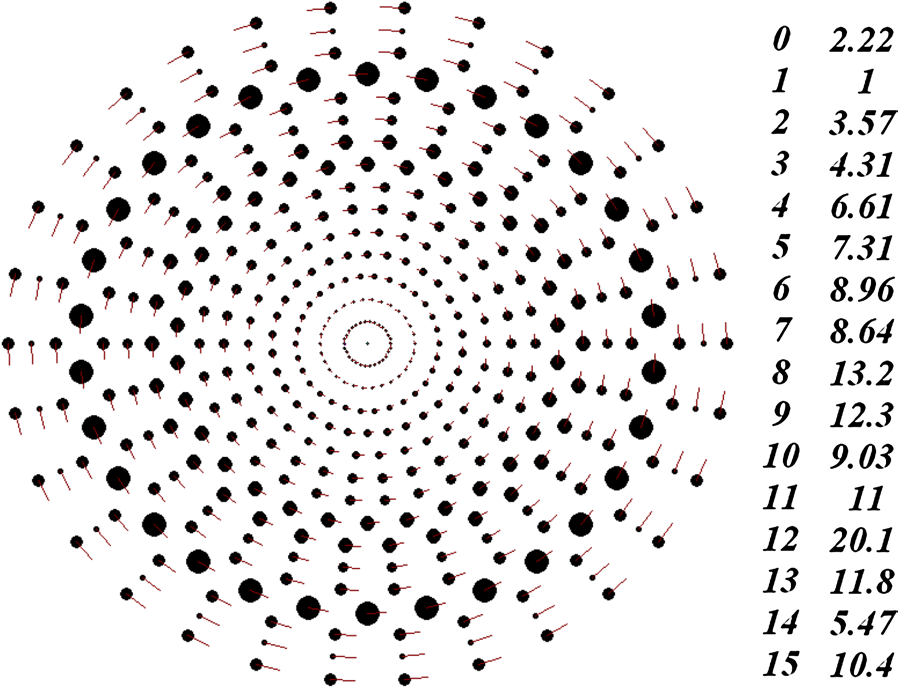
Multi-layer axisymmetric structure with *N*_2_ = 15 and *N*_3_ = 30; the angles *φ*_*j,*1_ = 0 and *φ*_*j,*1_ = 0.5·*Δφ*_0_ recur each three layers; *P*_*rd*_ = 1 year; *r*_*1*_ = 1.493837 × 10^11^ m, *m*_*1*_ = 8.889051 × 10^29^ kg and *m*_*SS*_ = 3.570164 × 10^33^ kg. For the rest designations, see Figure 2.

In the structure shown in Figure 4, the angle *φ*_*j,*1_ = *Δφ*_0_/2 recurs each three layers. Here, the total mass of the system has also increased in comparison with the radial-beam structure of Figure 2; yet, this mass proved to be lighter than the mass of the structure shown in Figure 3. The mass of the bodies in the rings also increases with increasing ring radius. In the rings with *φ*_*j,*1_ = *Δφ*_0_/2, the body masses are normally heavier than in the neighbor rings. The heaviest mass is in the ring with number 12. Among the three examples considered above, this mass is the heaviest one, being twenty times greater than the mass of the bodies in the first ring.

**Figure 5:**
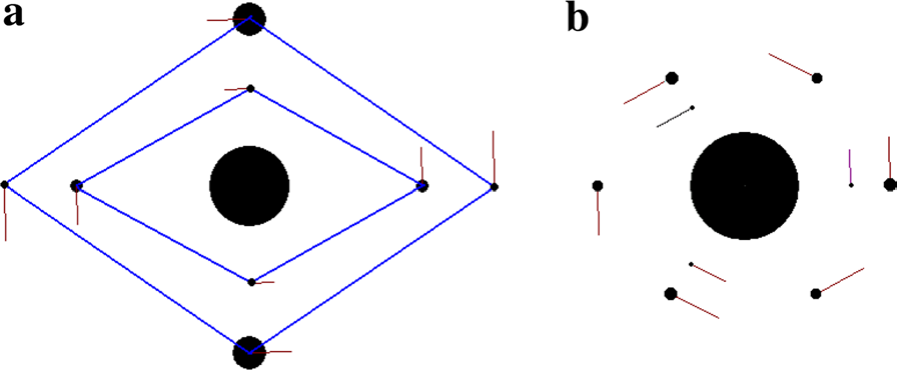
**a** The four-layer axisymmetric structure with *N*_2_ = 4 and *N*_3_ = 2; the angle *φ*_*j,*1_ = 0.5·*Δφ*_0_ alternates with the angle *φ*_*j,*1_ = 0; *P*_*rd*_ = 2 year; *r*_*1*_ = 1.491651 × 10^11^ m, *m*_*1*_ = 1.695854 × 10^27^ kg and *m*_*SS*_ = 4.373151 × 10^30^ kg. **b** The three-layer axisymmetric structure with *N*_2_ = 3 and *N*_3_ = 3; *φ*_*j,*1_ = 0, 0.5·*Δφ*_0_, and 0; *P*_*rd*_ = 1.5 year; *r*_*1*_ = 1.491990 × 10^11^ m, *m*_*1*_ = 9.018554 × 10^26^ kg and *m*_*SS*_ = 2.971883 × 10^30^ kg. The rest designations are the same as in Figure 2.

The majority of homographic problems are represented by mutually embedded regular polygons (Grebenikov 2010). On the other hand, irregular polygons such as, for instance, mutually embedded rhombs, are also met (Grebenikov 2010; Diarova and Zemtsova 2006). Such a rhombic multi-layer structure [see Figure 20 in (Grebenikov 2010)] can be constructed as a structure comprising several rings. In Figure 5a, this structure is shown as a structure comprising four rings with two bodies contained in each of the rings (*N*_2_ = 4 and *N*_3_ = 2). Here, the ring radii and the masses of one body in the rings normalized respectively by the radius of the first ring and by the mass of one body in this ring are *r*_*ud,j*_ = 0, 1, 1.697, 1.703 and 2.40, and *m*_*ud1,j*_ = 1,161, 1, 193.47, 490.54 and 23.76. Unlike in the previous structures, the angle of the first body in the first ring is *φ*_1,1_ = 0.5·*Δφ*_0_ = *π*/2 (see Figure 5a). That is why this body occupies position at the ordinate axis. That structure, comprising two rhombs, one rhomb being embedded into the other, is identical to the structure shown in Figure 20 of monograph (Grebenikov 2010).

As it was shown above, in the four-layer ring structure of Figure 5a the dimensionless radii of the second and third rings are roughly identical, *r*_*ud,3*_ = 1.697 and *r*_*ud,4*_ = 1.703. Nonetheless, the angles of the first body in those rings are shifted with respect to each other by the angle 0.5∙*Δφ*_0_. It follows from here that axisymmetric rotating structures having 2·*N*_3_ bodies in their separate rings can be constructed. For creating such structures, it is required that the radii of neighbor rings in them be identical while the angles of the first body in those rings, different. As an example, Figure 5b shows a three-layer structure with identical radii of the second and third layers. Here, the non-dimensional radii and the non-dimensional masses are respectively *r*_*ud,j*_ = 0, 1, 1.375 and 1.375, and *m*_*ud1,j*_ = 21,844, 1, 175.8 and 193.8. Evidently, here the radii of the second and third rings are identical while the masses of the bodies forming the rings are different. The latter structure is identical to the homographic configuration involving a regular hexagon which in turn contains a concentric equilateral triangle [see Figure 26 in monograph (Grebenikov 2010)].

The example in Figure 5b exhibits an interesting feature. Mathematically, this structure is defined as a structure that comprises three rings (*N*_2_ = 3), whereas, physically, here we have two rings, or two layers. Yet, the bodies in the second layer exhibit two possible mass patterns. Thus, the presented examples prove that a wide set of homographic configurations (Grebenikov 2010), or planar central configurations (Perko and Walter 1985), can be represented by multi-layer axisymmetric rotating structures.

As it was noted above, a structure comprising 103 rings and 29 bodies in each ring (*N*_2_ = 103 and *N*_3_ = 29) and a structure comprising 1,000 rings and 999 bodies in each ring (*N*_2_ = 1,000 and *N*_3_ = 999) were constructed. In the former structure, the total number of the bodies was *N* = 2,988, and in the latter structure this number was *N* = 999,001. In addition, structures with the starting mass *m*_*in*_ equal to the Earth’s mass were calculated. The total number of the rings in such structures was *N*_2_ = 6, 15, and 103. The generated files with input data and initial conditions for the program Galactica proved to be identical to the files obtained for an initial mass *m*_*in*_ somewhat heavier than the Sun’s mass. This means that the results obtained for the Sun’s mass (those results are shown in Figures 1, 2, 3, 4, 5) simultaneously present results for the structures whose central mass is equal to the Earth’s mass.

## Stability problems for examined structures and their applications

Despite the fact that the characteristics of examined structures were obtained as an exact solution of the problem, the solution results are inevitably expressed with numbers having a finite length of their representation with digits. As a result, the obtained characteristics of a structure obviously differ from the exact characteristics. The mentioned difference presents one factor that makes the structure change its configuration during numerical integration of the equations. The second factor is the precision of the integration method. If the structure is a stable one, then, with running time, the bodies making up the structure start executing low-amplitude oscillations being, as a rule, azimuthal oscillations. If the structure is unstable, then this structure finally suffers disintegration.

In the above-mentioned homographic-dynamics studies, especially in publication (Grebenikov 2010), problems on the stability of rotating structures were treated analytically within the framework of Hamiltonian dynamics. As it was noted in (Grebenikov 2010), there exist more than 100 definitions of the notion “stability”, and in space dynamics one can encounter up to thirty such definitions. Unfortunately, such methods fail to produce direct predictions for the evolution of particular structures. That is why, for predicting the dynamic evolution of a structure, it is required to perform numerical integration of differential motion equations for involved bodies. Here, the integration accuracy should be high enough, and the inaccuracy, small, so that to not distort the predicted behavior of the structure. All those requirements are met in the program Galactica.

**Figure 6:**
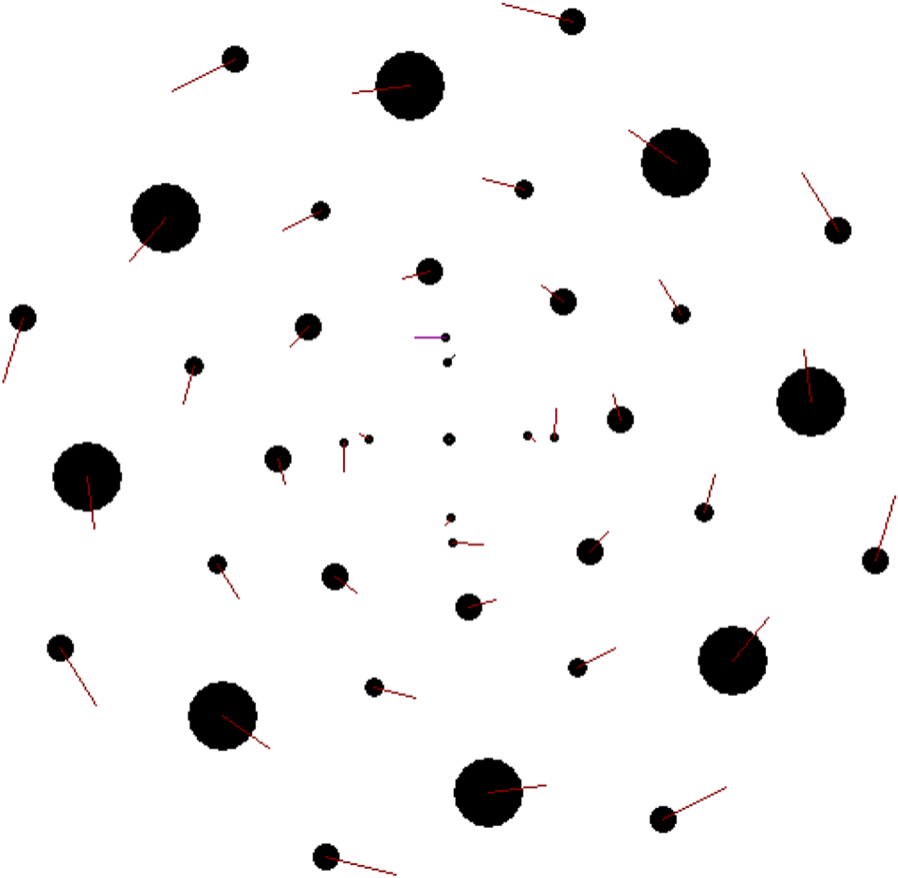
The onset of decomposition of the axisymmetric structure with *N*_2_ = 5, *N*_3_ = 8, and *P*_*rd*_ = 1 year in which the angle *φ*_*j,*1_ = 0 alternates with the angle *φ*_*j,*1_ = 0.5·*Δφ*_0_. The structure has executed 1.6 revolutions; the starting configuration of the structure is shown in Figure 1.

From the standpoint of the force interaction, all multi-layer rotating structures present unstable structures. Yet, the lifetime of such structures can vary over a wide range. Figure 6 shows the structure of Figure 1 after 1.6 revolutions. The changes in the structure have emerged because of the disintegration of the first, inner, ring. In this ring, the eight bodies have combined in pairs. Further motion of the paired bodies leads to a collapse of the whole structure.

The program Galactica was used to investigate the dynamics of axisymmetric structures in studies (Smulsky 2008, 2011, 2013b, 2014; Mel’nikov et al. 2008; Smul’skii 2011). Those studies have revealed a broad range of possible behaviors that could be displayed by axisymmetric structures. Of primary interest is the lifetime of investigated structures. This time is longer the lower is the non-dimensional rotational velocity of the system. If an axisymmetric structure presents a substructure of some greater structure, then the lifetime of the axisymmetric structure can increase in value. In the latter case, the bodies forming the structure execute oscillations as they move along their trajectories. Such oscillations act to prevent the bodies from approaching each other, and those oscillations may therefore infinitely prolong the lifetime of the structure (Mel’nikov et al. 2008).

Of special interest is using the program Galactica for performing studies of the dynamic properties of axisymmetric rotating structures. Such studies may lead to application of examined structures in various problems of celestial and space dynamics. For instance, single-layer axisymmetric structures were used in developing compound models for Earth and Sun’s rotation in (Smulsky 2008, 2011; Mel’nikov et al. 2008). In such studies, a qualitative picture for the evolution of Earth’s axis was revealed. In addition, the oscillations of peripheral bodies in such a model have suggested an idea of possible oscillating motion of Earth’s continents in latitude direction (Mel’nikov et al. 2008). The compound model for Sun’s rotation proved helpful in revealing the Mercury-perihelion excess rotation required for the development of a description of Solar-system dynamics based on the Newton law of gravitation.

Using the investigated structures for modeling planetary disks and disk galaxies also attracts interest. With body trajectories uniformly expanded in space, an axisymmetric structure becomes a spheroidal structure. Of special interest are the construction of such structures and the study of their dynamics. Very probably, new ideas in this field will prove helpful in gaining a better insight into the nature of globular star clusters and into the processes proceeding in such clusters.

## Conclusions

An exact solution to the problem of *N* bodies arranged in an in-plane multi-layer axisymmetric rotating structure has been obtained. The axial symmetry of such a structure consists in that the geometric and dynamic characteristics of the structure remain unchanged on its rotation through an angle *Δφ*_0_. Characteristics of particular structures with various numbers of involved layers and bodies (up to one million bodies) have been determined. The obtained characteristics were verified by numerical integration of the differential motion equations of the bodies forming the structures. Examination of the dynamic properties of the rotating structures with the help of the program Galactica will enable application of obtained data in various celestial- and space-mechanics models. The multi-layer rotating structures involve all the planar central configurations presently known in homographic dynamics.
